# Early commitment of cardiovascular autonomic modulation in Brazilian patients with congenital generalized lipodystrophy

**DOI:** 10.1186/s12872-017-0738-4

**Published:** 2018-01-12

**Authors:** Clarisse Mourão Melo Ponte, Virgínia Oliveira Fernandes, Maria Helane Costa Gurgel, Izabella Tamira Galdino Farias Vasconcelos, Lia Beatriz de Azevedo Souza Karbage, Christiane Bezerra Rocha Liberato, Carlos Antônio Negrato, Marília de Brito Gomes, Ana Paula Dias Rangel Montenegro, Renan Magalhães Montenegro Júnior

**Affiliations:** 1 Faculty of Medicine, Federal University of Ceará, Fortaleza, Brazil; 2 State University of Rio de Janeiro, Rio de Janeiro, Brazil; 3 Brazilian Society of Diabetes, São Paulo, Brazil

**Keywords:** Cardiovascular autonomic neuropathy, Lipodystrophy, Neuropathy complications, Diabetes, Insulin resistance, Leptin

## Abstract

**Background:**

Metabolic abnormalities in congenital generalized lipodystrophy (CGL) are associated with microvascular complications. However, the evaluation of different types of neuropathy in these patients, including the commitment of cardiovascular autonomic modulation, is scarce. The objective of the present study was to determine the prevalence of cardiovascular autonomic neuropathy (CAN) in patients with CGL compared with individuals with type 1 diabetes and healthy subjects.

**Methods:**

Ten patients with CGL, 20 patients with type 1 diabetes and 20 healthy subjects were included in the study. Controls were paired 1:2 for age, gender, BMI and pubertal stage. Heart rate variability (HRV) was analyzed using cardiovascular autonomic reflex tests, including postural hypotension test, Valsalva (VAL), respiratory (E/I) and orthostatic (30/15) coefficients, and spectral analysis of the HRV, determining very low (VLF), low (LF) and high (HF) frequencies components. The diagnosis of CAN was defined as the presence of at least two altered tests.

**Results:**

CAN was detected in 40% of the CGL patients, 5% in type 1 diabetes patients and was absent in healthy individuals (*p* < 0.05). We observed a significant reduction in the E/I, VLF, LF and HF in CGL cases vs. type 1 diabetes and healthy individuals and lower levels of 30/15 and VAL in CGL vs. healthy individuals. A significant positive correlation was observed between leptin and 30/15 coefficient (*r* = 0.396; *p* = 0.036) after adjusting for insulin resistance and triglycerides. Autonomic cardiovascular tests were associated with HbA1c, HOMA-IR, triglycerides and albumin/creatinine ratio in CGL cases.

**Conclusions:**

We observed a high prevalence of CAN in young patients with CGL, suggesting that insulin resistance, hypertriglyceridemia and hypoleptinemia, may have been involved in early CAN development. Additional studies are needed to evaluate the role of leptinemia in the physiopathogenesis of the condition.

**Electronic supplementary material:**

The online version of this article (10.1186/s12872-017-0738-4) contains supplementary material, which is available to authorized users.

## Background

Congenital generalized lipodystrophy (CGL) is a condition characterized by absence of subcutaneous adipose tissue, deposition of ectopic fat, and several metabolic alterations. This disease has an autonomic recessive inheritance and has an estimated prevalence of 1:10.000.000 live births, with approximately 300 to 500 cases described in the medical literature [1]. In Brazil, approximately 80 cases were reported in reference centers, the majority of which located in from the Northeast region [2–4]. Currently, four genes have been implicated in the diagnosis of the four subtypes of CGL (types 1 to 4): *AGPAT2*, *BSCL2*, *CAV1* (Caveolin1*)* and *PTRF* (polymerase I and transcript release factor). Each of these genes encodes proteins that play important roles in lipid homeostasis [1].

Reflecting a deficit of metabolic active adipose tissue, leptin deficiency impairs the metabolic activity and storage capacity of the subcutaneous adipose tissue, resulting in the accretion of ectopic fat in the liver and muscles. During the disease evolution, patients show hypertriglyceridemia, insulin resistance and a poorly controlled diabetes mellitus (DM), leading to early microvascular complications [1]. However, the evaluation of different types of neuropathy in these patients, including the evaluation of cardiovascular autonomic modulation, is scarce [3].

Most guidelines on diabetic neuropathy recommend the evaluation of heart rate variability (HRV) according to cardiovascular autonomic reflexes or Ewing’s tests (gold standard) for the diagnosis of cardiovascular autonomic neuropathy (CAN) [5]. Most recently, the determination of frequency domains (spectral analysis) and timing (statistical analysis) of HRV using specialized software has also been performed and have the advantage of not requiring patient cooperation [6].

Poor glycemic control and the duration of the disease are the most important risk factors for the occurrence of CAN, especially in patients with type 1 diabetes [7, 8]. Instead, in patients with type 2 diabetes, a combination of multiple factors accounts for the occurrence of CAN, such as hypertension, obesity, dyslipidemia and hyperglycemia [5]. Indeed, hyperglycemia alone does not explain the physiopathology of the neural lesion and among the factors potentially implicated in development of neuropathy, insulin resistance and abnormal lipid profile play an important role [9, 10].

These observations allow us to speculate that CGL could be an interesting biological model for the study of mechanisms that are potentially associated with the development of CAN in individuals exposed to an environment rich in severe metabolic abnormalities early in life. The aim of the present study was to compare the prevalence of CAN in CGL cases and type 1 diabetes patients using HRV tests and to evaluate the association between clinical and metabolic factors and CAN parameters in CGL patients.

## Methods

### Study population

The Brazilian Group for the Study of Inherited and Acquired Lipodystrophies (BrazLipo) from the University Hospital Walter Cantídio (Federal University of Ceará), a reference unit for follow-up of patients with CGL in Ceará State, performed a cross-sectional study from October 2013 to December 2015.

The CGL group comprised of 10 individuals who were regularly followed (2 to 3 visits per year), aged more than 7 years and able to cooperate with the tests performance. The initial sample of CGL patients followed during the study period included 15 individuals with ages varying from 1 to 30 years. Four cases younger than 7 years were excluded (2 males and 2 females) due non-compliance with cardiovascular reflexes tests, and in one case (18 years, ♀), were lost to follow-up. The presence of generalized lipodystrophy since birth or in the early stages of childhood was the main clinical criterion for the diagnosis of lipodystrophy. Other characteristics evaluated for diagnosis included the presence of acromegaloid facies, muscular hypertrophy, superficial hypertrophic veins (phlebomegaly), increased liver volume, hypertriglyceridemia and insulin resistance [11]. The description of the main clinical features of the patients with CGL is provided in the Table 1. Among the selected patients, seven patients had previously undergone a molecular diagnosis of CGL, with mutations in the *AGPAT2* gene in two patients (CGL type 1) and the *BSCL2* gene in five patients (CGL type 2) (described in the Additional file 1: Table S1).

**Table 1:** Clinical, metabolic, and genetics characterization of patients with congenital generalized lipodystrophy

Case (initials) Gender/Age Tanner	Subtype Mutation (gene)	Comorbidities, Microvascular complications	Drugs	CAN (yes/no)
1 (RMTS) ♀, 7 years M2P2	Type 2 CGL *BSCL2*	High HOMA-IR score, ↓HDL-c, ↑TG, nephropathy (moderate albuminuria), ↑BP	MTF	Yes (clinical)
2 (KEBS) ♀, 7 years M2P2	Type 1 CGL *AGPAT2*	High HOMA-IR score, ↓HDL-c, ↑TG	None	No
3 (JAGS) ♂, 9 years G1P1	DNA not available	High HOMA-IR score, ↓HDL-c, ↑TG	None	No
4 (ACLB) ♀, 10 years M2P2	Type 2 CGL *BSCL2*	DM, high HOMA-IR score, ↓HDL-c,↑TG	MTF, pioglitazone	Yes (incipient)
5 (LCS) ♂, 10 years G2P1	Type 2 CGL *BSCL2*	DM, ↓HDL-c, ↑TG,↑cholesterol, nephropathy (severe albuminuria)	MTF, pioglitazone, insulin	No
6 (DRM) ♂, 14 years G4P4	DNA not available	DM, ↓HDL-c, ↑TG, ↑cholesterol	MTF	No
7 (PS) ♀, 14 years M4P4	Type 2 CGL *BSCL2*	DM, ↓HDL-c, ↑TG, ↑cholesterol, nephropathy (severe albuminuria), peripheral neuropathy, ↑BP	MTF, acarbose, insulin, ciprofibrate	Yes (clinical)
8 (PCSF) ♂, 14 years G5P5	Type 2 CGL *BSCL2*	DM, high HOMA-IR score, ↓HDL-c, ↑TG, nephropathy (moderate albuminuria)	MTF	No
9 (BMS) ♀, 25 years M5P5	Type 1 CGL *AGPAT2*	DM, ↓HDL-c, ↑TG,↑cholesterol, ↑BP, nephropathy (severe albuminuria), peripheral neuropathy	MTF, pioglitazone, insulin, ciprofibrate	Yes (clinical)
10 (RMAS) ♀, 30 years M5P5	DNA not available	DM, ↓HDL-c, ↑TG, ↑BP, nephropathy (severe albuminuria)	MTF, insulin, losartan	Yes (clinical)

*CGL* congenital generalized lipodystrophy, *CAN* cardiovascular autonomic neuropathy, *HDL-c* high-density lipoprotein, *HOMA-IR* Homeostasis model assessment-insulin resistance, *TG* triglycerides, *BP* blood pressure, *DM* diabetes mellitus, *MTF* metformin

Twenty control subjects with type 1 diabetes were included from the database of Brazilian Type 1 Diabetes Study Group (BrazDiab1SG) [12]. Control subjects with type 1 diabetes were recruited at a ratio of 1:2 matched for age, gender, BMI, and pubertal stage. Subjects with T1 diabetes who were younger than 7 years, obese (BMI > 30), current use of drugs that could affect the cardiovascular system, use of anti-depressants or who were pregnant/lactating were excluded.

Twenty healthy individual volunteers were selected by phone call following the same criteria as for type 1 diabetes subjects in similar ratio. The exclusion criteria for this group were the same as for type 1 diabetes subjects adding the state of diabetes. Table 2 shows the clinical and biochemical parameters of the groups.

**Table 2:** Clinical and metabolic characterization of patients with congenital generalized lipodystrophy, type 1 diabetes and healthy individuals

Variables	CGL (*n* = 10)	Type 1 diabetes (*n* = 20)	Healthy (*n* = 20)	p1	p2	p3
Female, % (n)	60 (6)	55 (11)	60 (12)	1.000	1.000	1.000
Age (years)	12 (7; 30)	13 (7; 32)	12 (7; 31)	0.338	0.642	0.287
pBMI (%) children and adolescents	62 (41; 94) *n* = 8	69 (7; 93) *n* = 16	52 (10; 82) *n* = 16	0.602	0.100	0.175
BMI (Kg/m^2^) adults	22.3 (22.0; 22.7) *n* = 2	22.8 (21.2; 23.7) *n* = 4	23.7 (22.5; 24.8) *n* = 4	0.643	0.165	0.248
Age group	Pre-pubertal: 10 (1)	Pre-pubertal: 10 (2)	Pre-pubertal: 20 (4)	1.000	0.508	0.294
	Pubertal: 70 (7)	Pubertal: 70 (14)	Pubertal: 60 (12)			
	Adult: 20 (2)	Adult: 20 (4)	Adult: 20 (4)			
Basal HR (bpm)	90 (72; 109)	77 (59; 99)	71 (53; 94)	*0.013*	*0.006*	0.440
Systolic BP (mmHg)	123 (90; 175)	102 (87; 126)	104 (80; 113)	*0.004*	*0.001*	0.694
Dyastolic BP (mmHg)	78 (50; 109)	66 (50; 86)	66 (60; 80)	*0.004*	*0.006*	0.479
Diabetes Mellitus, % (n)	70 (7)	100 (20)	0	*0.030*	*0.000*	*0.000*
Diabetes duration (years)	8 (1; 14)	5 (1; 12)	0	NA	0.260	NA
Glycated hemoglobin (mmol/mol)	55 (25; 109)	62 (33; 105)	33 (22; 40)	*0.251*	*0.006*	*0.000*
Glycated hemoglobin (%)	7.2 (4.4; 12.1)	7.8 (5.2; 11.8)	5.2 (4.2; 5.8)	*0.251*	*0.006*	*0.000*
Peripheral neuropathy, % (n)	30 (3)	15 (3)	0	0.372	*0.030*	0.231
Nephropathy, % (n)	60 (6)	0	0	*0.000*	*0.000*	1.000
Metformin, % (n)	80 (8)	0	0	*0.000*	*0.000*	1.000
Pioglitazone, % (n)	30 (3)	0	0	*0.030*	*0.030*	1.000
Losartan, % (n)	10 (1)	0	0	0.333	0.333	1.000
Ciprofibrate, % (n)	20 (2)	0	0	0.106	0.106	1.000
Use of insulin, % (n)	40 (4)	100 (20)	0	*0.000*	*0.008*	*0.000*
Fasting plasma glucose (mmol/l)	5.4 (3.8; 13,8)	6,2 (4,2; 12,3)	4.5 (4.7; 5.2)	*0.644*	*0.045*	*0.000*
Basal insulin (mUI/mL)	27.8 (6.7; 102.0)	NA	9.8 (2.8; 13.4)	NA	*0.000*	NA
HOMA-IR	6.8 (1.2; 9.3)	NA	2.2 (0.5; 2.8)	NA	*0.000*	NA
Total Cholesterol (mmol/l)	3.5 (2.5; 20.0)	NA	4.0 (2.1; 5.1)	NA	0.644	NA
HDL-cholesterol (mmol/l)	0.8 (0.6; 1.3)	NA	1.4 (1.2; 2.2)	NA	*0.000*	NA
LDL-cholesterol (mmol/l)	2.1 (1.2; 4.0)	NA	2.2 (1.1; 3.7)	NA	0.611	NA
Triglycerides (mmol/l)	1.3 (1.0; 80.4)	NA	0.8 (0.5; 1.7)	NA	*0.001*	NA
Leptin (ng/mL)	1.1 (0.8; 1.7)	NA	4.9 (1.3; 33.0)	NA	*0.000*	NA
ACR (mg/g)	85.3 (3.1; 5535.0)	NA	5.9 (2.5; 24.9)	NA	*0.004*	NA

*CGL* congenital generalized lipodystrophy, *BMI* body mass index, *HR* cardiac frequency, *BP* blood pressure, *DM* diabetes mellitus, *HOMA-IR* Homeostasis model assessment-insulin resistance, *HDL* high-density lipoprotein, *LDL* low-density lipoprotein, *ACR* albumin/creatinine ratio, *NA* not available. Tests: Fischer’s exact test for categorical variables and Mann-Whitney test for continuous variables; p1: comparison between CGL and type 1 diabetes groups; p2: comparison between CGL and healthy groups; p3: comparison between type 1 diabetes and healthy individuals. Statistical significance *p* < 0.050 are marked in italic

The present study was approved by the ethics committee of University Hospital of Ceará Federal University. Written informed consent was obtained from all participants or their parents prior to inclusion.

### Study protocol

All individuals were interviewed, and all of the clinical parameters were evaluated, including the use of medicines, presence of microvascular complications (diabetic nephropathy and neuropathy), presence of hypoglycemia and symptoms of dysautonomia (dizziness, syncope or pre-syncope, resting tachycardia, polyuria, urinary urgency, urine retention, urinary incontinence, sexual dysfunction, constipation, diarrhea, fecal incontinence, nausea, vomiting, postprandial fullness, plenitude postprandial, and gustatory sweating). To obtain these data, in addition to the interview, the principal investigator conducted medical chart reviews.

Anthropometric measurements and arterial blood pressure were obtained following specific recommendations [13]. Tanner’s pubertal classification was used to determine the pubertal stage [14]. DM was diagnosed based on the American Diabetes Association criteria [15]. Hypertriglyceridemia and the reduction of HDL-cholesterol were defined according to the recommendations of the National Cholesterol Education Program Adult Treatment Panel [16], regarding age and gender. The presence of peripheral neuropathy was assessed using the Neuropathy Total Symptom Score (TSS) [17] and the Neuropathy Disability Score (NDS) [18]. The presence of nephropathy was evaluated based on the albumin/creatinine ratio (ACR) in an isolated urine sample performed on two different occasions, as well as the glomerular filtration rate (GFR), classified according to specific recommendations (ACR between 30 and 300 mg/g: moderate albuminuria; ACR ≥ 300 mg/g: severe albuminuria) [19].

### Laboratory analysis

All blood and urine samples were correctly identified and drawn after a 12-h fast. The blood samples were centrifuged at 3.000 rpm for 10 min to separate serum from plasma. Subsequently, the samples were stored in a freezer at −80 °C until further analysis. Biochemical evaluation was performed through the determination of glycemia, total cholesterol, HDL-cholesterol, triglycerides, creatinine and ACR using an enzymatic colorimetric method according to the manufacturer’s instructions. Glycated hemoglobin (HbA1c) was dosed by high performance liquid chromatography (PREMIER®–Trinity Biotech). Insulin was determined by electrochemiluminescence (HITACHI®–Roche). Leptin was dosed using an enzyme immunoassay (AIKA®–Diasorin; REF: CAN-L-4260; sensibility: 0.5 ng/mL; variation coefficient intra-assay: 3.7 to 5.0%). The Homeostasis model assessment-insulin resistance (HOMA-IR) was calculated using the following equation: HOMA-IR = fasting glycemia (mmol = mg/dl ÷ 18) x fasting insulinemia (μU/ml)/22.5 [20]. Elevated HOMA-IR was considered for scores higher than 3.43 in children and adolescents [21] and higher than 2.7 in individuals older than 18 years [22].

### Cardiovascular autonomic tests

Cardiovascular autonomic reflexes tests, spectral analysis (frequency domain) and statistical analysis (time domain) of the HRV were performed in order to evaluate cardiovascular autonomic modulation, based on a digital electrocardiogram (Poly-Spectrum System, version 4.8.143.0, Russia).

Individuals were evaluated in fasting state during the morning. Capillary glycemia was determined prior to the beginning of the test and had to be in the range of 70 and 250 mg/dL. The patients were asked not to inject rapid-acting insulin at least two hours prior to beginning of the tests, to avoid the use of caffeine for at least eight hours before the test and to not vigorously exercise for 24 h before the evaluation. Volunteers presenting with a fever (temperature ≥ 37.8 °C) in the previous two days, important emotional stress in the previous day or hypoglycemia at 8 h before the tests were asked to postpone testing.

The tests were started at 15 min after a resting with the patient lying down and a head inclination of 30 degrees. After this time interval, a 300-s electrocardiogram was performed. The electrocardiogram was analyzed using a mathematical algorithm (Fourier transformation) and expressed in a diagram with amplitude oscillations (cardiac frequency fluctuations per second) versus the cardiac frequency (hertz) (spectral analysis). The total amplitude of HRV comprised three bands: (1) very low frequency component VLF (0.01–0.04 Hz), (2) low frequency component or LF (0.04–0.15 Hz) and (3) high frequency component or HF (0.15–0.5 Hz). The relation between LF/HF was calculated, reflecting the sympathetic-vagal balance and total spectrum balance. Statistical analysis of HRV was performed by using standard deviation of the average of the RR interval (SDNN) and the square root of the RR interval average (SDRMS).

After the evaluation of the HRV spectral and statistical analyses, cardiovascular autonomic reflexes tests were performed. A resting period of 1 min was maintained between tests to prevent influences from previous tests.

1. Deep breathing test (E/I coefficient): during the electrocardiogram, the volunteer has performed deep inspiration and expiration with at least a 5 s duration each. The duration of each respiratory cycle was signaled from the volunteer to the researcher. The E/I coefficient was obtained by dividing the longest RR interval [minimal heart rate (HR) during expiration] by the shortest RR interval (maximal HR during inspiration). Each respiratory cycle was repeated three times, and the best ratio obtained was considered to be the final result.
2. Valsalva maneuver: during the electrocardiogram, the volunteer performed a breathing exercise to maintain a pressure of 40 mmHg that was evaluated using a manometer for 15 s. At approximately 14 s, a maximal physiologic tachycardia was expected to occur. After this exercise, deep breathing was interrupted, and the electrocardiogram register as maintained for 45 s, while a physiologic bradycardia was expected to occur. The Valsalva coefficient was obtained according to the relation between the longest (minimal HR) and shortest (maximal HR) RR intervals. The occurrence of a facial flushing, plethora and cervical veins engorgement indicated that the test was adequately performed. The best out of two results was considered the definitive result.
3. Orthostatic test (30/15 coefficient): after rest in supine position, the volunteer had to remain in an orthostatic position, and the relation between the RR interval corresponding to the maximal bradycardia around the 30th heart beat and the maximal tachycardia around the 15th heart beat after orthostatic position was considered to be the final result.
4. Orthostatic or postural hypotension test: after a 30 min rest in supine position, the arterial blood pressure was evaluated and compared with the pressure at 3 min after the beginning of the orthostatic position. A drop equal to or higher than 20 mmHg in the systolic blood pressure was considered to be altered.

The same researcher performed all of the tests, and all of the electrocardiograms were reviewed to determine whether a sinus cardiac rhythm was present and to exclude the presence of arrhythmias or any other confounding factor. The intra-individual reproducibilities of the respiratory and Valsalva’s coefficients were evaluated, observing elevated correlations (*r* = 0.775; *p* < 0.001, and *r* = 0.806; p < 0.001, respectively).

To evaluate the results, reference values for age were used. The cut off points for HRV tests were according to Ziegler et al. (1992) and Spallone et al. (2011) [5, 23]. The respiratory (E/I) and orthostatic (30/15) coefficients, HF component, and time domains were considered to evaluate the parasympathetic autonomic nervous system (ANS); the postural hypotension test and the VLF component were considered to evaluate the sympathetic ANS; Valsalva’s coefficient, LF component, total amplitude spectrum, and the LF/HF ratio were considered to simultaneously evaluate the sympathetic and parasympathetic ANS. Clinical CAN was diagnosed in the presence of at least two altered HRV tests, and incipient CAN was diagnosed in the presence of only one altered test. The presence of postural hypotension confirmed the diagnosis of advanced CAN [5, 24].

### Statistical analysis

Data were analyzed using the Statistical Package of Social Science (SPSS Inc., Chicago, IL, USA), version 15.0 for Windows and R3.3.1 software. Continuous variables were described as the mean and median (minimum; maximum), and categorical variables were described according to the relative and absolute frequencies. The Mann-Whitney test was used for continuous variables. Associations between categorical variables were analyzed using the chi-square test and Fisher’s exact test. For the correlation analysis, the correlation Spearman test was used. The Bootstrap technique was used for the estimation of the confidence interval of the Spearman coefficient for the analysis of the CGL group (*n* = 10). A *p*-value less than 0.050 were considered statistically significant.

## Results

**Fig. 1:**
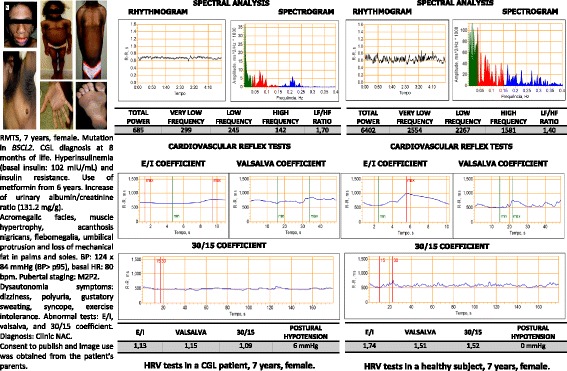
Evaluation tests for the cardiac frequency variation in a patient with congenital generalized lipodystrophy and a healthy control. LF/HF: low frequency/high frequency; 30/15: orthostatic coefficient; E/I: respiratory coefficient; HRV: heart rate variability test; CGL: congenital generalized lipodisthrophy

Clinical CAN was observed in 40% (4/10) of patients with CGL, 5% (1/20) of patients with type 1 diabetes and none of the healthy individuals. CAN was observed as an impairment of the parameters of the sympathetic and parasympathetic cardiovascular autonomic system of patients with CGL compared to patients with type 1 diabetes and healthy individuals (Table 3). Figure 1 shows the tests results for the evaluation of HRV in a patient with CGL and a healthy individual.

**Table 3:** Prevalence of cardiovascular autonomic neuropathy and parameters for the evaluation of cardiovascular parasympathetic and sympathetic nervous system in patients with congenital generalized lipodistrophy, type 1 diabetes and healthy individuals

Variables	CGL (n = 10)	Type 1 diabetes (n = 20)	Healthy (n = 20)	p1	p2	p3
Clinical CAN % (n)	40 (4)	5 (1)	0	*0.031*	*0.008*	1.000
Incipient CAN % (n)	10 (1)	15 (3)	0	1.000	0.333	0.115
CAN % (n)	50 (5)	20 (4)	0	0.115	*0.002*	0.106
Altered 30/15 coefficient % (n)	40 (4)	15 (3)	0	0.181	*0.008*	0.231
Altered Valsalva coefficient % (n)	10 (1)	0	0	0.333	0.333	1.000
Altered E/I coefficient % (n)	30 (3)	5 (1)	0	0.095	*0.030*	1.000
Postural hypotension % (n)	10 (1)	0	0	0.333	0.333	1.000
*Parasympathetic System*						
30/15 coefficient	1.19 (0.98; 1.59)	1.34 (1.04; 1.68)	1.55 (2.26; 2.02)	0.064	*0.001*	*0.001*
E/I coefficient	1.33 (1.09; 1.57)	1.52 (1.14; 2.01)	1.60 (1.23; 2.23)	*0.004*	*0.001*	0.323
Component of high frequency (Hz)	627 (55; 1840)	1447 (182; 8374)	2993 (270; 9969)	*0.019*	*0.001*	0.064
SDNN	64 (22; 169)	60 (25; 124)	82 (36; 163)	0.965	0.113	*0.039*
RMSSD	57 (12; 237)	52 (17; 170)	85 (18; 180)	0.877	0.252	0.069
*Sympathetic system*						
Reduction in SBP >10 mmHg % (n)	30 (3)	10 (2)	5 (1)	0.300	0.095	1.000
Reduction in SBP > 20 mmHg % (n)	10 (1)	0	0	0.333	0.333	1.000
Component of very low frequency (Hz)	383 (88; 4250)	1077 (392; 5857)	1988 (688; 18,341)	*0.017*	*0.002*	*0.026*
*Parasympathetic and sympathetic systems*						
Valsalva coefficient	1.54 (1.15; 2.34)	1.70 (1.24; 2.52)	1.74 (1.50; 2.42)	0.166	*0.039*	0.297
Component of low frequency (Hz)	329 (139; 2525)	972 (181; 3109)	1916 (343; 5580)	*0.033*	*0.001*	*0.017*
Total spectrum amplitude (Hz)	1582 (501;9691)	4482 (755; 12,854)	7125 (1853; 24,788)	*0.019*	*0.000*	*0.013*
LF/HF ratio	1.21 (0.12; 3.70)	0.67 (0.19; 1.99)	0.58 (0.26; 1.70)	0.235	0.454	0.892

*CGL* congenital generalized lipodystrophy, *CAN* cardiovascular autonomic neuropathy, 30/15 orthostatic coefficient, *E/I* respiratory coefficient, *SDNN* standard deviation of the RR interval average, *RMSSD* square root of the RR interval average, *SBP* systolic blood pressure, *LF/HF* Low frequency/high frequency component. Tests: Fischer’s exact test; Mann-Whitney test; p1: comparison between CGL and type 1 diabetes groups; p2: comparison between CGL and healthy groups; p3: comparison between type 1 diabetes and healthy individuals. Statistical significance *p* < 0.050 are marked in italic

The frequency of CAN in patients with and without nephropathy was 67% (4/6) and 25% (1/4); *p* = 0,524, respectively. Patients with CGL presented a higher frequency of postural hypotension in relation to the healthy group (30% vs. 0%; *p* = 0.030); a difference between the other dysautonomia symptoms was not observed. A description of the results of the tests in patients with CGL and the symptoms in each group are individually described in the Additional file 1: Tables S2 and S3.

When the clinical parameters and cardiovascular autonomic tests were compared in a subgroup of patients with lipodystrophy and DM (*n* = 7) to a subgroup of patients with type 1 diabetes (*n* = 14) after matching for age, gender, glycemic control and duration of diabetes, higher values of HR and systolic blood pressure and lower levels of the respiratory coefficient, VLF and LF components and total spectrum amplitude among the patients with lipodystrophy and DM were observed (Additional file 1: Table S4).

The autonomic tests presented an association with several metabolic parameters (Table 4). A positive correlation between leptin and the 30/15 coefficient (*r* = 0.396; *p* = 0.036), was observed, even after controlling for the effect of the variables HOMA-IR and triglycerides. The time domains SDNN and SDRMS did not show a correlation with the metabolic parameters.

**Table 4:** Correlations between cardiovascular autonomic tests and metabolic parameters of patients with CGL and healthy individuals

*n* = 30		30/15	Valsalva	E/I	VLF	LF	HF	Total espectrum
Age	r	0.155	−0.245	−0.190	−0.029	−0.128	−0.275	−0.181
	p	0.412	0.191	0.313	0.876	0.499	0.142	0.337
BMI	r	0.034	−0.182	−0.447	−0.201	−0.315	−0.436	−0.405
	p	0.860	0.335	*0.013*	0.287	0.090	*0.016*	*0.026*
Fasting glycemia	r	−0.204	−0.184	−0.253	−0.488	−0.313	−0.259	−0.311
	p	0.280	0.330	0.178	*0.006*	0.093	0.167	0.095
A1c	r	−0.400	−0.190	−0.310	−0.460	−0.525	−0.326	−0.434
	p	*0.029*	0.315	0.096	*0.010*	*0.003*	0.079	*0.017*
Basal Insulin	r	−0.432	−0.358	−0.610	−0.501	−0.590	−0.645	−0.583
	p	*0.017*	0.052	*0.000*	*0.005*	*0.001*	*0.000*	*0.001*
HOMA-IR	r	−0.439	−0.362	−0.591	−0.543	−0.561	−0.598	−0.555
	p	*0.015*	*0.049*	*0.001*	*0.002*	*0.001*	*0.000*	*0.001*
Leptin	r	0.537	0.312	0.291	0.338	0.339	0.379	0.364
	p	*0.002*	0.094	0.119	0.067	0.067	*0.039*	*0.048*
ACR	r	−0.514	−0.200	−0.356	−0.627	−0.374	−0.276	−0.477
	p	*0.004*	0.289	0.053	*0.000*	*0.042*	0.140	*0.008*
Total cholesterol	r	−0.259	−0.021	−0.262	−0.103	−0.193	−0.145	−0.156
	p	0.167	0.913	0.161	0.589	0.306	0.444	0.411
HDL-c	r	0.366	0.248	0.301	0.313	0.198	0.199	0.221
	p	*0.046*	0.186	0.106	0.092	0.294	0.292	0.240
LDL-c	r	−0.259	0.058	−0.214	−0.027	−0.079	−0.123	−0.077
	p	0.184	0.769	0.275	0.892	0.688	0.532	0.698
Triglycerides	r	−0.632	−0.261	−0.674	−0.470	−0.548	−0.420	−0.539
	p	*0.000*	0.163	*0.000*	*0.009*	*0.002*	*0.021*	*0.002*

*CGL* congenital generalized lipodystrophy, *BMI* body mass index, *A1c* glycated hemoglobin, *HOMA-IR* Homeostasis model assessment-insulin resistance, *HDL* high-density lipoprotein, *LDL* low-density lipoprotein, *ACR* albumin/creatinine ratio, 30/15 orthostatic coefficient, E*/I* respiratory coefficient, *VLF* very low frequency, *LF* low frequency, *HF* high frequency. Test: Spearman’s correlation posts. Significance *p* < 0.050 are marked in italic

When the CGL group was analyzed alone, the 30/15 coefficient showed an inverse correlation with the triglyceride levels (*r* = −0.778; *p* = 0.008) and the E/I coefficient showed an inverse correlation with ACR (*r* = −0.769; *p* = 0.009) and triglycerides (*r* = −0.818, *p* = 0.004). The LF component was inversely correlated with HbA1c (*r* = −0.636; *p* = 0.048), ACR (*r* = −0.063; p = 0.048) and triglycerides (*r* = −0.648; *p* = 0.042), and the LF/HF ratio showed a positive correlation with HOMA-IR (*r* = 0.673; *p* = 0.033). After the *Bootstrap* reassembly technique was applied in CGL group, the variables that maintained the correlation included the E/I coefficient and triglycerides (IC95%: -1.000; −0.036) and ACR (IC95%: -0.945; −0.301); LF component and triglycerides (IC95%: -0.950; −0.198); and LF/HF ratio and HOMA-IR (IC95%: 0.231; 0.884). A correlation between serum leptin levels and results of the autonomic tests was not observed in this group.

## Discussion

To the best of our knowledge, this is one of the first study to evaluate the prevalence of CAN in CGL patients. We have followed a standardized protocol and employed gold-standard method for the clinical analysis. Half of the patients were diagnosed with a rare CGL presenting alterations of the autonomic cardiovascular function. This may confirm the precocity and severity of the microvascular complications observed in these patients.

Faria et al. (2009), in Rio Grande do Norte state (Brazil), evaluated 18 patients with CGL (age: 21.3 ± 8.3 years) to analyze the HRV time domain (statistical analysis) according to a 24-h electrocardiogram. The results showed lower values of the SDNN and SDRMSS time domains in patients with CGL vs. the healthy group matched for gender and age, with no association between the metabolic parameters and cardiovascular autonomic tests [3]. These parameters are not typically considered to be diagnostic criteria for CAN in the published guidelines in the literature [5, 24].

In the present study, the cardiovascular autonomic tests revealed an association with the values of HOMA-IR. In recent years, insulin resistance has been implicated in the pathophysiology of diabetic neuropathy, and several studies have shown the presence of CAN in patients with pre-diabetes and metabolic syndrome, independent of the presence of hyperglycemia [25–27]. In a previous study conducted with more than 1200 patients with metabolic syndrome, a high prevalence of CAN was observed in patients with one or more alterations that are generally present in cases of metabolic syndrome [28]. In the Finnish Diabetes Prevention Study, the diagnostic parameters of CAN were associated with higher triglycerides levels and a larger waist circumference [29]. Insulin per se increases serum catecholamine levels as well as reduces parasympathetic control of HR and is therefore associated with the development of CAN [30].

Nevertheless, the role of hyperinsulinemia in this scenario has been debated for many years, and some authors have questioned whether cardiovascular autonomic dysfunction, with an impairment of the parasympathetic system and activation of ANS, proceeds the occurrence of insulin resistance [31, 32]. In patients without lipodystrophy, the impairment of the cardiovascular autonomic function contributes to the pathophysiology of insulin resistance. Sympathetic activation inhibits insulin secretion by pancreatic beta cells. Vasoconstriction mediated by sympathetic action reduces muscle glucose uptake as a result of blood flux reduction and an increase in lipolysis in adipose tissue. This lipolysis leads to an increase in the production of circulating free fatty acids (FFAs), which increase insulin resistance, thereby maintaining a vicious circle [28]. The results of the present study, however, suggest that insulin resistance proceeds and is associated with lesions in nerves involved in autonomic modulation, considering that insulin resistance is the background metabolic abnormality of CGL and is present very early in patients with this disease. Notwithstanding, in the case of CGL, the presence of an autonomic sympathetic activation may contribute to the worsening of insulin resistance in these patients.

In the present study, triglyceride levels were associated with the parameters for the evaluation of HRV. Clinical and epidemiological studies have shown a strong association between dyslipidemia, particularly hypertriglyceridemia and neuropathy in patients with type 1 diabetes and type 2 diabetes [33]. FFAs can cause lipotoxicity mediated through lysosomal dysfunction in neuron and Schwann’s cell cultures [34]. Animal studies have shown that a fat-rich diet leads to the deposition of sorbitol, oxidation of lipids and activation of lipoxygenases in peripheral nerves, independent of the development of hyperglycemia [35]. Experimental studies with cell culture have suggested that subclinical inflammation and oxidative and nitrosative stress promote the activation of neural apoptosis through mitochondrial dysfunction and endoplasmic reticulum stress [36]. Furthermore, an increase in the FFA circulating levels can have systemic effects, such as increased inflammatory cytokines, leading to neuronal inflammation and the eventual development of neuropathy [34]. Thus, a deficit in subcutaneous fat deposition as observed in CGL and an increase in circulating FFAs levels can contribute to an adverse environment that is favorable for the development of neuronal damage in these patients.

It has been suggested that leptin has an independent action over cardiovascular autonomic modulation. For example, in obesity, high leptin levels are associated with the activation of the sympathetic ANS, as a compensatory mechanism against weight gain [27]. Higher levels of leptin have also been observed in obese patients with impairment in cardiovascular autonomic modulation, as evaluated by HRV tests [37]. A recent systematic analysis showed that high leptin levels were associated with diabetic neuropathy, including autonomic neuropathy, independent of BMI and diabetes duration [38].

The consequences of severe hypoleptinemia on the ANS are not well understood. A priori, hypoleptinemia leads to the inhibition of the sympathetic ANS. Considering the inhibitory effect of low serum leptin levels on the sympathetic system associated with its impairment that occurs during the evolution of neuropathy itself, these patients present an even more favorable scenario for the development of the most severe types of CAN. Leptin stimulates neurogenesis, axonal growth, synaptogenesis and dendritic cells, both pre and post birth. The neuroprotection actions of leptin include the inhibition of apoptosis and improvement of cell survival through regulation of apoptotic enzymes (inhibition of Bcl-xL expression, caspases and TRAIL ligands and activation of neurotrophic factors synthesis), protection against glutamatergic cytotoxicity as well as against oxidative stress through the expression of anti-oxidant factors, stabilization of the mitochondrial membrane and stimulation of progenitor cells [39]. These leptin actions were observed only in the central nervous system, however, we can speculate that this activity could also occur in the autonomic system, and the severe leptin deficiency observed in patients with CLG could contribute, among many factors, to the development of more severe forms of CAN. In the present study, we observed a positive correlation between leptin levels and autonomic tests in patients with CGL and healthy individuals after adjusting for insulin resistance and triglycerides, and the association was not observed when patients with CGL were studied separately. The small sample and extremely low leptin levels may indicate a bias in the analysis. This may be mentioned that correlation analysis do not necessarily suggest a causality.

A higher but non- significant prevalence of CAN was observed among patients with nephropathy. This is likely to have ensued due to low sample size. In addition, a correlation between the ACR values and cardiovascular autonomic tests was also observed. Several clinical and epidemiological trials have shown that CAN is associated with a higher risk of diabetic nephropathy. The increase in sympathetic tonus observed in the initial phases of CAN could impair renal homeostasis, blood flux and the filtration and excretion of sodium, accelerating the progression to renal dysfunction [40, 41]. Moreover, CAN promotes a loss of nocturnal decrease in blood pressure, a factor that is closely associated with microalbuminuria and chronic renal disease [42]. These findings could explain the high prevalence of nephropathy among patients with CGL.

Patients with CAN present a higher frequency of dysautonomia symptoms; however, a significant difference only for the presence of postural hypotension (dizziness, syncope or pre-syncope when standing) was observed. The clinical features of CAN are unspecific, poorly associated with abnormalities in autonomic tests and observed only in the advanced stages of the disease and therefore do not present adequate sensibility or specificity for the diagnosis of CAN. Notably, postural hypotension is associated with the presence of a more severe neuropathy and higher rates of cardiovascular mortality, reflecting a high prognostic value, and should be routinely evaluated in patients with DM, even in individuals who do not present symptoms [24].

We could not compare CGL in patients with type 1 and type due to limited sample size. The majority of patients had type 2 CGL, characterized by the presence of null mutations (with protein function loss) in the *BSCL2* gene*.* These patients showed more severe metabolic complications. This finding may reflect a more severe impairment in the storage function of lipids drops in the adipocytes of patients with seipin deficiency, which is responsible for the fusion of lipids drops and adipocyte differentiation [1]. Notably, a group of neurodegenerative diseases is associated with the presence of *missense* mutations in *BSCL2* (associated with protein’s function improvement). These diseases have been described and generically named seipinopathies [43, 44]. Moreover, some patients with type 2 CGL show neurologic manifestations, such as spastic contractures, superior motor neuron’s disease, and a precocious and fatal neurodegenerative syndrome [1]. However, in the present study, no patients with type 2 CGL showed any of these neurological alterations and no mutations in *BSCL2* which have been previously described in patients with type 2 CGL with these manifestations.

The cardiovascular reflexes tests used in the present study showed high sensibility and specificity (80 to 90%) as well as good reproducibility for the diagnosis of CAN [45, 46]. Previous studies have shown a variation coefficient lower than 10% for deep breathing and orthostatic tests and a coefficient between 10 and 15% for the Valsalva maneuver [47]. Although there is no evidence for the superiority of one test in relation to others [48], the Valsalva’s test presents higher sensitivity for the detection of earlier phases of CAN because it evaluates both the sympathetic and parasympathetic systems [24]. In addition to the gold-standard tests, examination of the HRV frequency (VLF, LF, HF) and time domains using specialized software showed high (99%) and specificity (100%) and were not dependent on patient cooperation [49]. We emphasize that the VLF component has been recommended less often since its interpretation in short recordings (5 min or less) is less clear [50] and this could be a study limitation. However, this parameter was used in combination to the others HRV tests and CAN diagnosis was done according to recommended in CAN Subcommittee of the Toronto Consensus Panel Statement [24]. In addition, the two patients who presented alteration of VLF component had criteria for CAN diagnosis due to alterations in the others HRV tests, minimizing the impact of this limitation.

## Conclusion

We observed a high prevalence of CAN in young patients with CGL compared to type 1 diabetes patients. This may suggest that the metabolic abnormalities observed in CGL, including insulin resistance, hypertriglyceridemia and hypoleptinemia, may have been involved in early CAN development. However, additional studies are needed to evaluate the role of leptinemia in the physiopathogenesis of this condition.

## Additional file

Additional file 1: Table S1. Molecular results of the AGPAT2 and BSCL2 genes in patients with generalized congenital lipodystrophy. **Table S2.** Results of the cardiovascular autonomic tests of patients with congenital generalized lipodystrophy. **Table S3.** Symptoms of dysautonomia in patients with congenital generalized lipodistrophy and type 1 diabetes as well as healthy individuals. **Table S4.** Evaluation of the cardiometabolic parameters and cardiovascular autonomic tests in patients with lipodystrophy and type 1 diabetes. (DOCX 36 kb)

## Abbreviations

30/15: Orthostatic coefficient
ACR: Albumin-creatinine ratio
ANS: Autonomic nervous system
BMI: Body mass index
BP: Blood pressure
BrazDiab1SG: Brazilian type 1 diabetes study group
CAN: Cardiovascular autonomic neuropathy
CGL: Congenital generalized lipodysthrophy
DM: Diabetes mellitus
E/I: Respiratory coefficient
FFA: Free fatty acids
GFR: Glomerular filtration rate
HbA1c: Glycated hemoglobin
HDL: High-density lipoprotein
HF: High frequency
HOMA-IR: Homeostasis model assessment-insulin resistance
HR: Heart rate
HRV: Heart rate variability
LDL: Low-density lipoprotein
LF: Low frequency
NDS: Neuropathy Disability Score
SDNN: Standard deviation of the average of the RR interval
SDRMS: Square root of the RR interval average
TSS: Neuropathy Total Symptom Score
VAL: Valsalva coefficient
VLF: Very low frequency
