# Supplementary material for: Early commitment of cardiovascular autonomic modulation in Brazilian patients with congenital generalized lipodystrophy
Source: BMC Cardiovasc Disord. 2018 Jan 12;18:6. doi: 10.1186/s12872-017-0738-4 (PMC5767058; doi:10.1186/s12872-017-0738-4)
Supplement: Additional file 1: Table S1. — Molecular results of the AGPAT2 and BSCL2 genes in patients with generalized congenital lipodystrophy. Table S2. Results of the cardiovascular autonomic tests of patients with congenital generalized lipodystrophy. Table S3. Symptoms of dysautonomia in patients with congenital generalized lipodistrophy and type 1 diabetes as well as healthy individuals. Table S4. Evaluation of the cardiometabolic parameters and cardiovascular autonomic tests in patients with lipodystrophy and type 1 diabetes. (DOCX 36 kb) [file 12872_2017_738_MOESM1_ESM.docx]

**ADDITIONAL FILES**

**ADDITIONAL FILES S1**

| Table S1 – Molecular results of the AGPAT2 and BSCL2 genes in patients with generalized congenital lipodystrophy. | | | | | |
| --- | --- | --- | --- | --- | --- |
| **Case (initials)** | **Gender**  **Age (years)** | **Gene** | | **Mutation** | **ID** |
| **1** (**RMTS)** | Fem, 7 | *BSCL2* | Éxon4 | c.325dupA, p.Thr109Asnfs*5 | rs786205071 |
| **2 (KEBS)** | Fem, 7 | *AGPAT2* | Éxon3 | c.369_372deLGCTC, p.Leu124Serfs*26 | Mutação nova |
| **3 (JAGS)** | Masc, 9 | - | - | - | - |
| **4 (ACBL)** | Fem, 10 | *BSCL2* | Éxon4 | c.412C>T, p.Arg138* | rs137852970 |
| **5 (LCS)** | Masc, 10 | *BSCL2* | Éxon4 | c.325dupA, p.Thr109Asnfs*5 | rs786205071 |
| **6 (DRM)** | Masc, 14 | - | - | - | - |
| **7 (PS)** | Fem, 14 | *BSCL2* | Éxon4 | c.325dupA, p.Thr109Asnfs*5 | rs786205071 |
| **8 (PCSFJ)** | Masc, 14 | *BSCL2* | Éxon4 | c.301_302insAA, p.Met101Lysfs*11 | rs786205069 |
| **9 (BMS)** | Fem, 25 | *AGPAT2* | Éxon3  Íntron4 | c.369_372deLGCTC, p.Leu124Serfs*26  c.589-2A>G | Mutação nova  rs116807569 |
| **10 (RMAS)** | Fem, 30 | - | - | - | - |

**ADDITIONAL FILES S2**

| Table S2 – Results of the cardiovascular autonomic tests of patients with congenital generalized lipodystrophy. | | | | | | | | | |
| --- | --- | --- | --- | --- | --- | --- | --- | --- | --- |
| **Case (initials)**  **Mutation** | **30/15** | **E/I** | **Valsal-va** | **Reduction in SBP (mmHg)** | **VLF (Hz)** | **LF (Hz)** | **HF (Hz)** | **Altered tests** | **CAN** |
| **1 (RMST)**  **Type 2 CGL**  ♀ 7 years | 1.09 | 1.13 | 1.15 | < 10 | 299 | 245 | 142 | 30/15  E/I;  Valsalva | Clinical |
| **2 (KEBS)**  **Type 1 CGL**  ♀ 7 years | 1.57 | 1.57 | 1.64 | < 10 | 4250 | 4294 | 1147 | Ø | Absent |
| **3 (JAGS)**  **NA**  ♂ 9 years | 1.19 | 1.39 | 1.63 | < 10 | 933 | 742 | 663 | Ø | Absent |
| **4 (ACBL)**  **Type 2 CGL**  ♀ 10 years | 1.13 | 1.36 | 1.45 | < 10 | 88 | 158 | 652 | 30/15;  Valsalva | Incipient |
| **5 (LCS)**  **Type 2 CGL**  ♂ 10 years | 1.25 | 1.27 | 2.34 | < 10 | 327 | 229 | 1840 | Ø | Absent |
| **6 (DRM)**  **NA**  ♂ 14 years | 1.19 | 1.34 | 1.74 | < 10 | 1437 | 694 | 1243 | Ø | Absent |
| **7 (PS)**  **Type 2 CGL**  ♀ 14 years | 0.98 | 1.21 | 1.25 | 10 a 20 | 394 | 388 | 329 | 30/15; E/I;  Valsalva | Clinical |
| **8 (PCSFJ)**  **Type 2 CGL**  ♂ 14 years | 1.59 | 1.46 | 1.82 | < 10 | 694 | 756 | 603 | Ø | Absent |
| **9 (BMS)**  **Type 1 CGL**  ♀ 25 years | 0.99 | 1.09 | 1.41 | ≥ 20 | 306 | 139 | 55 | 30/5; E/I;  Valsalva;  LF; HF; postural hypotension | Clinical /  Advanced |
| **10 (RMSA)**  **NA**  ♀ 30 years | 1.21 | 1.33 | 1.29 | 10 a 20 | 203 | 271 | 147 | Valsalva;  VLF | Clinical |
| Legend – CGL: congenital generalized lipodystrophy; 30/15: orthostatic coefficient; E/I: respiratory coefficient; SBP: systolic blood pressure; VLF: very low frequency; LF: low frequency; HF: high frequency; CAN: cardiovascular autonomic neuropathy NA: not available. | | | | | | | | | |

**ADDITIONAL FILES S3**

| Table S3 – Symptoms of dysautonomia in patients with congenital generalized lipodistrophy and type 1 diabetes as well as healthy individuals. | | | | | | |
| --- | --- | --- | --- | --- | --- | --- |
| **Variables** | **CGL**  **(n=10)** | **Type 1 diabetes**  **(n=20)** | **Healthy**  **(n=20)** | **p1** | **p2** | **p3** |
| **Genitourinary tract**  (pollakiuria, urinary urgency, retention or incontinence, sexual dysfunction) | 40 (4) | 20 (4) | 10 (2) | 0.384 | 0.141 | 0.661 |
| **Lower gastrointestinal tract**  (constipation, diarrhea or fecal incontinence) | 0 | 20 (4) | 5 (1) | 0.272 | 1.000 | 0.342 |
| **Upper gastrointestinal tract**  (nausea, early satiety, vomit) | 0 | 0 | 25 (5) | 0.140 | - | 0.047 |
| **Gustatory sweating** | 20 (2) | 20 (4) | 0 | 1.000 | 0.103 | 0.106 |
| **Postural hypotension**  (dizziness, syncope) | 30 (3) | 20 (4) | 0 | 0.657 | **0.030** | 0.106 |
| **Total** | 60 (6) | 40 (8) | 15 (3) | 0.442 | **0.030** | 0.155 |
| Legend – CGL: congenital generalized lipodystrophy. Tests: Fischer’s exact; p1: comparison between CGL and type 1 diabetes groups; p2: comparison between CGL and healthy groups; p3: comparison between type 1 diabetes and healthy individuals. Statistical significance p < 0.05. | | | | | | |

**ADDITIONAL FILES S4**

| Table S4 – Evaluation of the cardiometabolic parameters and cardiovascular autonomic tests in patients with lipodystrophy and type 1 diabetes. | | | |
| --- | --- | --- | --- |
| **Variables** | **Lipoatrofic diabetes**  **(n=7)** | **Type 1 diabetes**  **(n=14)** | **p** |
| **Female, % (n)** | 57,1 (4) | 50 (7) | 1.000 |
| **Age (years)** | 14 (10; 30) | 14 (11; 32) | 0.706 |
| **pBMI (%) children and adolescents** | 58 (41; 86)  n=5 | 72 (8; 93)  n=10 | 0.854 |
| **BMI (Kg/m²) adults** | 22.3 (22.0; 22.7)  n=2 | 22.8 (21.2; 23.7)  n=4 | 0.643 |
| **DM duration (years)** | 8 (1; 14) | 5 (2; 12) | 0.260 |
| **Glycated hemoglobin (mmol/mol)** | 61 (44; 109) | 62 (37; 105) | 0.940 |
| **Glycated hemoglobin (%)** | 7.7 (6.2; 12.1) | 7.8 (5.5; 11.8) | 0.940 |
| **Insulin dose (units/kg)** | 4.8 (1.3; 5.6) | 1.1 (0.4; 0.5) | **0.029** |
| **Basal HR (bpm)** | 86 (81; 109) | 79 (59; 99) | **0.004** |
| **SBP (mmHg)** | 130 (110; 175) | 104 (96; 126) | **0.002** |
| **DBP (mmHg)** | 80 (50; 109) | 70 (50; 86) | 0.117 |
| **CAN, % (n)** | 57,1 (4) | 42,8 (6) | 0.659 |
| **Clinical CAN % (n)** | 42.9 (3) | 7.1 (1) | 0.088 |
| **30/15 Coefficient** | 1.19 (0.98; 1.59) | 1.33 (1.04; 1.58) | 0.135 |
| **Valsalva’s coefficient** | 1.45 (1.25; 2.34) | 1.64 (1.24; 2.06) | 0.737 |
| **E/I Coefficient** | 1.33 (1.09; 1.46) | 1.52 (1.14; 2.01) | **0.001** |
| **Drop on BP (mmHg)** | 8 (0; 22) | 6 (0; 12) | 0.177 |
| **Very low frequency (Hz)** | 372 (88; 1437) | 1033 (392; 5857) | **0.009** |
| **Low frequency (Hz)** | 271 (139; 756) | 972 (181; 3109) | **0.015** |
| **High frequency (Hz)** | 603 (55; 1840) | 1193 (182; 8374) | 0.073 |
| **Total spectrum (Hz)** | 1111 (501; 3374) | 3763 (755; 12854) | **0.025** |
| **LF/HF ratio** | 1.18 (0.12; 2.50) | 0.67 (0.30; 1.89) | 0.455 |
| **SDNN (ms)** | 61 (22; 169) | 57 (25; 124) | 0.794 |
| **RMSSD (ms)** | 81 (16; 237) | 49 (17; 170) | 0.390 |

Legend – DM: diabetes mellitus; BMI: body mass index; HR: cardiac frequency; BP: blood pressure; CAN: cardiovascular autonomic neuropathy; 30/15 coefficient: orthostatic coefficient; E/I coefficient: respiratory coefficient; LF/HF: low frequency/high frequency component; SDNN: RR interval standard deviation; RMSSD: square root of the average RR interval. Tests: Fisher’s exact test for categorical variables and Mann-Whitney test for continuous variables. Statistical significance p < 0.05.
